# Safety and Effectiveness of Vibegron in Japanese Patients With Overactive Bladder: A Japanese Postmarketing Survey

**DOI:** 10.1111/luts.12535

**Published:** 2024-11-05

**Authors:** Shoko Yoshimura, Hiromitsu Yagi, Kazunori Abe, Masakazu Yamasaki

**Affiliations:** ^1^ Pharmacovigilance Department Kyorin Pharmaceutical Co. Ltd. Tokyo Japan; ^2^ Pharmacovigilance & Post‐Marketing Surveillance Kissei Pharmaceutical Co. Ltd. Tokyo Japan

**Keywords:** β_3_‐adrenoceptor agonist, overactive bladder, postmarketing survey, vibegron

## Abstract

**Objectives:**

To evaluate the safety and effectiveness of vibegron, a highly selective β_3_‐adrenoceptor agonist, in Japanese patients with overactive bladder (OAB) in a real‐world clinical setting.

**Methods:**

This prospective survey was conducted from August 2019 to July 2023 using a central registration method. Patients newly treated with vibegron for OAB were followed for 12 weeks, and those who continued treatment were observed for up to 52 weeks. Adverse drug reactions (ADRs) and the overall improvement level were assessed, and overactive bladder symptom score (OABSS) and International Prostate Symptom Score‐Quality of Life (IPSS QOL) score were collected.

**Results:**

Of the 1848 patients in the safety analysis set, 154 ADRs were reported in 141 patients (7.63%). Common ADRs were increased residual urine volume (1.30%), constipation (1.14%), dysuria and urinary retention (0.97% each), cystitis (0.65%), and dry mouth (0.32%). The incidence of urinary retention‐related adverse events (AEs) was higher in male patients, patients ≥ 65 years old, and patients with benign prostatic hyperplasia. The effectiveness rate was 88.8% among the 1561 patients in the effectiveness analysis set. Significant improvement in both OABSS and IPSS QOL scores was observed at all observation periods. The rate of achieving the minimal clinically important change in OABSS total score at the final evaluation was 75.35%. OABSS scores were also significantly reduced in all subgroup analyses.

**Conclusions:**

This survey found no major concerns regarding the safety and effectiveness of vibegron in Japanese OAB patients, except for urinary retention‐related AEs. Vibegron is considered to represent a useful drug for treating OAB symptoms in a real‐world clinical setting.

## 1 Introduction

Overactive bladder (OAB) is a symptom complex defined as “urgency, with or without urge incontinence, usually with frequency and nocturia.” [1] This common chronic condition has a significant negative impact on the quality of life of those affected [2, 3]. Several large‐scale epidemiological studies have found prevalences of OAB in adults exceeding 10% in Japan and Western countries [4, 5, 6]. In addition, numbers of OAB patients are expected to increase as populations around the world continue to age. The mainstay of pharmacotherapy over the past decades has been oral anticholinergic agents, but these are associated with poor rates of persistence and adherence because of the limited efficacy and undesirable class‐related adverse events (AEs), such as dry mouth and constipation [7, 8, 9].

Vibegron, a β_3_‐adrenoceptor agonist, has been approved in multiple countries at different doses (50 or 75 mg). The target receptor, the β_3_‐adrenoceptors, is highly expressed in the detrusor muscle of the human bladder and is mainly involved in relaxing detrusor smooth muscle fibers during the filling stage of the micturition cycle [10, 11, 12]. Vibegron relaxes the bladder by selectively acting on β_3_‐adrenoceptors, increasing the bladder capacity [13, 14]. β_3_‐Adrenoceptor agonists are better tolerated than anticholinergic agents because they do not have anticholinergic AEs and concerns with β_3_‐adrenoceptor agonists on heart rate or blood pressure are much less often seen and less likely to be associated with drug discontinuation [15]. In comparison to mirabegron, the first β_3_‐adrenoceptor agonist to gain approval in the world, vibegron has the advantages of requiring no warnings for patients of reproductive age and showing no contraindications for pregnant or breastfeeding patients. Moreover, while mirabegron is partially metabolized by cytochrome P450 (CYP) 3A4 and inhibits CYP2D6, vibegron exhibits no inhibitory or inducing effects on CYP isoenzymes such as CYP3A4 and CYP2D6, reducing the risk of drug–drug interactions [13].

In clinical trials in Japan, the United States, and Korea, vibegron was shown to be effective in improving symptoms of OAB and was tolerated extremely well in terms of safety [16, 17, 18]. Posthoc analysis of a Phase 3 study in Japan showed the efficacy of this drug against nocturia and urgency urinary incontinence, as well as cardiovascular safety [19, 20, 21]. Vibegron, however, is administered to patients with various backgrounds in a real‐world clinical setting, including patients with benign prostatic hyperplasia (BPH) and cardiovascular diseases as comorbidities and patients taking α_1_ blockers as concomitant medications, and information on these patients was limited in clinical trials. The present survey was therefore conducted to evaluate the safety and effectiveness of vibegron in a real‐world clinical setting in Japan.

## 2 Methods

This general‐use results survey was performed in accordance with the Japanese Ministerial Ordinance on Good Postmarketing Study Practice from the Ministry of Health, Labour, and Welfare [22, 23, 24].

### 2.1 Survey Design and Patients

This survey was conducted prospectively from August 2019 to July 2023 using a central registration method. To confirm the occurrence of urinary retention, which is an important identified risk in the Japanese risk management plan, the target sample size was set at 1200 based on a 0.88% incidence of urinary retention‐related adverse drug reactions (ADRs) in Japanese Phase III trials. Case registration and case report forms (CRFs) were completed by investigating physicians using an electronic data‐capturing system. Investigating physicians’ registered cases within 2 weeks from the date of starting vibegron. Patients who had been newly administered vibegron for the approved indications of urgency, increased urinary frequency, or urgency urinary incontinence associated with OAB were targeted and followed for 12 weeks, with patients who were continuing treatment at 12 weeks observed up to 52 weeks. Diagnostic criteria for OAB were defined as an overactive bladder symptom score (OABSS) urinary urgency score ≥ 2 and a total score ≥ 3.

### 2.2 Data Collection

The following information was collected: patient background (date of birth [age], sex, presence/absence of pregnancy, height, weight, inpatient/outpatient status, medical history, comorbidities, indications for vibegron, and date of onset of OAB), daily dose and duration of vibegron treatment, previous OAB medications, concomitant medications, combination treatment, residual urine volume, prostate volume, adverse events (AEs), results of laboratory tests related to AEs, overall improvement level, and clinical symptoms (OABSS score, International Prostate Symptom Score‐Quality of Life [IPSS QOL] score). AEs and comorbidities were adopted as reported by the investigating physicians and there were no unified diagnostic criteria among cases.

### 2.3 Safety Evaluation

ADRs were defined as AEs for which a causal relationship to vibegron could not be ruled out. All AEs were aggregated according to the event terms reported by investigating physicians using system organ class (SOC) and preferred term (PT) descriptions based on the Japanese version of the International Council for Harmonization's Medical Dictionary for Regulatory Activities (MedDRA/J) version 26.0. Seriousness was classified as serious or nonserious according to the Enforcement Regulation of the Pharmaceutical and Medical Device Act. The same PT event occurring multiple times in the same patient was only counted as one event. PT urinary retention includes “residual urine” or “feeling of residual urine” as its subterms. In this survey, “urinary retention‐related events” were defined to include events classified as PT urinary retention, PT dysuria, and PT residual urine volume increased.

### 2.4 Effectiveness Evaluation

Overall improvement level was comprehensively judged as “effective,” “ineffective,” or “not evaluable” by investigating physicians based on clinical symptoms at 52 weeks after starting vibegron or at the time administration was discontinued. “Not evaluable” cases were excluded from the effectiveness analysis set (EAS). The ratio of “effective” cases in the EAS was calculated as the effectiveness rate.

OABSS was evaluated at baseline and each observation period (less than 12 weeks, 12 weeks or more but less than 26 weeks, 26 weeks or more but less than 52 weeks, 52 weeks or more, and at final evaluation). The minimal clinically important change (MCIC) score was defined as a decrease of three points in the OABSS total score [25]. IPSS QOL score was an index expressing patient satisfaction with their current urinary status, evaluated using a 7‐point scale (0–6 points) for the same observation periods used for OABSS assessments. If multiple scores were available for each observation period for both OABSS and IPSS QOL scores, scores from the later date were used.

### 2.5 Statistical Analysis

Within‐group comparisons were performed using Fisher's exact test, and the Cochran–Armitage test was used when category order had to be considered. The Wilcoxon signed‐rank test was used to test for differences from baseline in OABSS and IPSS QOL scores in each observation period. The level of significance was set at 5% or less on both sides.

## 3 Results

### 3.1 Patient Disposition and Baseline Characteristics

**FIGURE 1:**
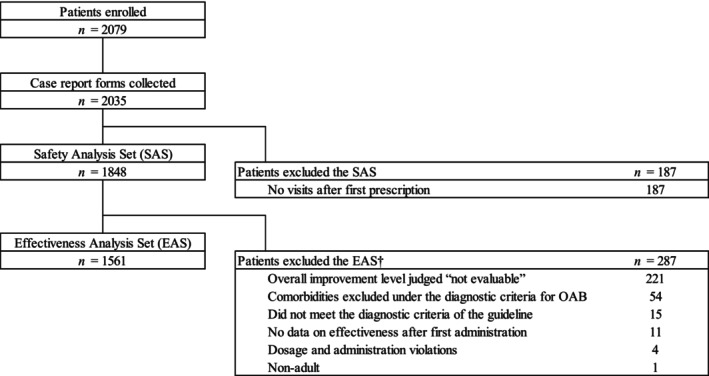
Patient disposition. †Including duplicated patients. OAB, overactive bladder.

In total, 2079 patients were enrolled from 416 medical institutions across the country, and 2035 CRFs were collected. Among those 2035 cases, 1848 patients were included in the safety analysis set (SAS) after excluding 187 patients with no visits after the first prescription (Figure 1). The EAS included 1561 patients after excluding another 287 patients from the SAS.

In the SAS, 500 patients completed or discontinued treatment within the 12‐week observation period, 563 patients received treatment for more than 12 weeks but less than 52 weeks and 785 patients received treatment for 52 weeks or more. By 12 weeks, 70 patients completed treatment for symptom remission (14.0%), and treatment was discontinued for the reasons of no further visits after an initial follow‐up visit (47.6%, 238 patients), persistent or aggravated symptoms (15.0%, 75 patients), ADRs (13.0%, 65 patients), and patient request (6.4%, 32 patients). Among the 563 patients who received treatment for 12 weeks or more but less than 52 weeks, 159 patients completed treatment for symptom remission (28.2%) and others discontinued treatment for the reasons of no further visits after a follow‐up visit (39.6%, 223 patients), patient request (9.1%, 51 patients), persistent or aggravated symptoms (8.9%, 50 patients), and ADRs (6.6%, 37 patients).

Baseline characteristics of the SAS (*n* = 1848) are shown in Table 1. Males accounted for 44.5% (822 patients) and 1423 patients (77.0%) were ≥ 65 years old. The severity of OAB as determined by the OABSS total score as mild (OABSS ≤ 5), moderate (6 ≤ OABSS ≤ 11) and severe (12 ≤ OABSS) at baseline was mild in 194 patients (10.5%), moderate in 1337 patients (72.3%), and severe in 305 patients (16.5%). The mean residual urine volume was 19.74 ± 24.454 mL and 169 patients (9.1%) had a residual urine volume ≥ 50 mL. Among male patients, the mean prostate volume was 34.14 ± 14.244 mL and 63 patients (7.7%) had a prostate volume ≥ 50 mL. Previous medications for OAB including β_3_‐adrenoceptor agonists (16.7%, 304 patients) and anticholinergic agents (9.2%, 168 patients) were reported in 437 patients (23.6%). Patients with comorbidities accounted for 63.6%, with those comorbidities including lower urinary tract obstruction disease in 479 patients (26.6%) and cardiovascular disease in 28.6% (516 patients). Concomitant medications were used in 1141 patients (61.7%), including α_1_ blockers (21.3%, 390 patients), phosphodiesterase (PDE) 5 inhibitors (3.7%, 67 patients), and anticholinergic agents (6.1%, 112 patients).

**TABLE 1:** Patient background of the safety analysis set (SAS) and incidence of urinary retention–related adverse events by background factors.

			*n* (%)	Patients with urinary retention–related AEs (%)	*p*
Patients in the safety analysis set (SAS)			1848 (100.0)	57 (3.08)	
Sex	Male		822 (44.5)	39 (4.74)	Fisher's exact test *p* < 0.001
	Female		1026 (55.5)	18 (1.75)	
Age category 1	< 54 years		205 (11.1)	1 (0.49)	Cochran–Armitage test *p* < 0.001
	≥ 55, < 65 years		220 (11.9)	1 (0.45)	
	≥ 65, < 75 years		534 (28.9)	15 (2.81)	
	≥ 75, < 85 years		711 (38.5)	32 (4.50)	
	≥ 85 years		178 (9.6)	8 (4.49)	
Age category 2	< 65 years		425 (23.0)	2 (0.47)	Fisher's exact test *p* < 0.001
	≥ 65 years		1423 (77.0)	55 (3.87)	
Age category 3	< 75 years		959 (51.9)	17 (1.77)	Fisher's exact test *p* < 0.001
	≥ 75 years		889 (48.1)	40 (4.50)	
Prostate volume (males only)	< 20 mL		26 (3.2)	0 (0.00)	Cochran–Armitage test *p* = 0.041
	≥ 20, < 30 mL		165 (20.1)	13 (7.88)	
	≥ 30, < 40 mL		114 (13.9)	4 (3.51)	
	≥ 40, < 50 mL		53 (6.4)	3 (5.66)	
	≥ 50 mL		63 (7.7)	10 (15.87)	
	Unknown		401 (48.8)	9 (2.24)	
Previous OAB medication	No		1388 (75.1)	40 (2.88)	Fisher's exact test *p* = 0.343
	Yes		437 (23.6)	17 (3.89)	
	Unknown		23 (1.2)	0 (0.00)	
	β3–adrenoceptor agonist	No	1521 (83.3)	44 (2.89)	Fisher's exact test *p* = 0.206
		Yes	304 (16.7)	13 (4.28)	
	Anticholinergic agents	No	1657 (90.8)	49 (2.96)	Fisher's exact test *p* = 0.237
		Yes	168 (9.2)	8 (4.76)	
Comorbidities	No		629 (34.0)	10 (1.59)	Fisher's exact test *p* = 0.006
	Yes		1175 (63.6)	46 (3.91)	
	Unknown		44 (2.4)	1 (2.27)	
Comorbidities category	Lower urinary tract obstruction disease	No	1325 (73.4)	25 (1.89)	Fisher's exact test *p* < 0.001
		Yes	479 (26.6)	31 (6.47)	
	Cardiovascular disease	No	1288 (71.4)	39 (3.03)	Fisher's exact test *p* = 0.764
		Yes	516 (28.6)	17 (3.29)	
Comorbidities details	Benign prostatic hyperplasia (males only)	No	343 (42.2)	8 (2.33)	Fisher's exact test *p* = 0.004
		Yes	470 (57.8)	31 (6.60)	
Concomitant medications	No		686 (37.1)	12 (1.75)	Fisher's exact test *p* = 0.008
	Yes		1141 (61.7)	45 (3.94)	
	Unknown		21 (1.1)	0 (0.00)	
Concomitant medications category	α1–antagonists	No	1437 (78.7)	33 (2.30)	Fisher's exact test *p* < 0.001
		Yes	390 (21.3)	24 (6.15)	
	Phosphodiesterase (PDE) 5 inhibitors	No	1760 (96.3)	50 (2.84)	Fisher's exact test *p* = 0.003
		Yes	67 (3.7)	7 (10.45)	
	Anticholinergic agents	No	1715 (93.9)	50 (2.92)	Fisher's exact test *p* = 0.081
		Yes	112 (6.1)	7 (6.25)	
Concomitant medications details	Tamsulosin hydrochloride	No	1680 (92.0)	49 (2.92)	Fisher's exact test *p* = 0.129
		Yes	147 (8.0)	8 (5.44)	
	Silodosin	No	1683 (92.1)	44 (2.61)	Fisher's exact test *p* < 0.001
		Yes	144 (7.9)	13 (9.03)	
	Naftopidil	No	1739 (95.2)	54 (3.11)	Fisher's exact test *p* = 0.752
		Yes	88 (4.8)	3 (3.41)	
	Tadalafil	No	1760 (96.3)	50 (2.84)	Fisher's exact test *p* = 0.003
		Yes	67 (3.7)	7 (10.45)	
	Dutasteride	No	1762 (96.4)	46 (2.61)	Fisher's exact test *p* < 0.001
		Yes	65 (3.6)	11 (16.92)	
	Fesoterodine fumarate	No	1789 (97.9)	55 (3.07)	Fisher's exact test *p* = 0.333
		Yes	38 (2.1)	2 (5.26)	
Combination treatment	No		1632 (88.3)	56 (3.43)	Fisher's exact test *p* = 0.016
	Yes		203 (11.0)	1 (0.49)	
	Unknown		13 (0.7)	0 (0.00)	
Combination treatment details	Pelvic floor exercises	No	1726 (94.1)	56 (3.24)	Fisher's exact test *p* = 0.254
		Yes	109 (5.9)	1 (0.92)	
	Bladder training	No	1736 (94.6)	56 (3.23)	Fisher's exact test *p* = 0.365
		Yes	99 (5.4)	1 (1.01)	

Abbrevaition: OAB, overactive bladder.

### 3.2 Safety

Among the 1848 patients in the SAS, 154 ADRs were reported in 141 patients (7.63%) (Table 2). Urinary retention‐related ADRs, namely, urinary retention (including events reported as “residual urine” or “feeling of residual urine”), residual urine volume increased, and dysuria occurred in 56 patients (3.03%). Of the 56 patients, residual urine volume increase was developed in 24 patients (1.30%), and urinary retention and dysuria were developed in 18 patients (0.97%) each. Regarding the administration period, the incidence of ADRs was significantly higher in less than 2 weeks, while there was not much difference in more than 2 weeks (Table 3). No signals of ADRs specific to long‐term use were observed.

**TABLE 2:** Incidence of adverse drug reactions.

	Patients, *n* (%)
Patients in the safety analysis set (SAS)	1848
Number of patients with ADRs (%)	141 (7.63)
Number of patients with serious ADRs (%)	9
Common ADRs by PT observed at 0.3% or higher	
Residual urine volume increased	24 (1.30)
Constipation	21 (1.14)
Dysuria	18 (0.97)
Urinary retention	18 (0.97)
Cystitis	12 (0.65)
Dry mouth	6 (0.32)

*Note:* MedDRA/J version 26.0.

Abbreviations: ADRs, adverse drug reactions; PT, preferred term.

**TABLE 3:** Incidence of adverse drug reactions by background factors by adverse drug reactions.

		*n* (%)	Patients with ADRs (%)	*p*
Patients in the safety analysis set (SAS)		1848 (100.0)	141 (7.63)	
Administration period of vibegron	< 2 weeks	57 (3.1)	22 (38.60)	Cochran–Armitage test *p* < 0.001
	≥ 2, < 4 weeks	140 (7.6)	15 (10.71)	
	≥ 4, < 12 weeks	303 (16.4)	30 (9.90)	
	≥ 12, < 26 weeks	331 (17.9)	25 (7.55)	
	≥ 26, < 52 weeks	232 (12.6)	24 (10.34)	
	≥ 52 weeks	785 (42.5)	25 (3.18)	

Of the 154 ADRs, 10 serious ADRs were reported in 9 patients, comprising 2 cases of hospitalization, and 1 case each of death, alopecia, onychomadesis, pyelonephritis acute, breast cancer, intestinal obstruction, cholelithiasis, and urinary retention. Alopecia and onychomadesis were reported in the same patient. Outcomes were “not recovered” for alopecia and onychomadesis, “unknown” for two hospitalizations and the case of breast cancer, and “recovered or recovering” for the remaining serious ADRs Regarding the case death, the family of the patient reported that he had died 89 days after discontinuing vibegron. The cause of death was unknown. The investigating physician determined that there was no causal relationship with vibegron.

AEs related to urinary retention, which is an important identified risk in the Japanese risk management plan of vibegron, were observed in 57 patients (3.08%, Table 1). One case of urinary retention was classified as serious, but resolved while continuing vibegron. Of the 18 patients developing urinary retention, 6 patients required indwelling urethral catheterization and 5 patients required a urethral catheter just to remove the retained urine. All of them recovered or improved after the treatment and discontinuation of vibegron. The 57 patients with urinary retention‐related AEs included 39 males (68.4%), of whom 31 patients had BPH reported by investigating physicians and 22 patients were using α_1_ blockers. No characteristic trends were observed regarding comorbidities or concomitant medications among the 18 female patients with urinary retention‐related AEs.

Potentially contributory background factors were examined in the 57 patients with urinary retention‐related AEs. The incidence of urinary retention‐related AEs was significantly higher among male patients, patients ≥ 65 years old, patients with a prostate volume ≥ 50 mL, patients with lower urinary tract obstruction disease or BPH as comorbidity, or patients using α_1_ blockers or PDE5 inhibitors as concomitant medications (*p* < 0.05).

The incidence of urinary retention‐related ADRs in patients with or without concomitant BPH was analyzed (Table 4). Concomitant BPH was identified in 470 of the 822 (57.2%) male patients in the SAS. The incidence of urinary retention‐related ADRs was 6.60% (31 patients) in the 470 patients with BPH and 2.33% (8 patients) in the 343 patients without BPH. Of the 31 patients with BPH who experienced urinary retention‐related ADRs, 29 patients were receiving treatment for BPH. The incidence of urinary retention‐related ADRs was 6.82% among patients being treated for BPH and 4.65% among patients without BPH treatment.

**TABLE 4:** Incidence of ADRs related to urinary retention in patients with BPH.

BPH and treatment status	No BPH		With BPH		Treatment status for BPH						BPH unknown		Total	
					No		Yes		Unknown					
Patients, *n* ^a^	343		470		43		425		2		9		822	
Patients with ADRs, *n*	8		31		2		29		0		0		39	
ADRs, *n*	9		34		2		32		0		0		43	
Incidence of ADRs, %	2.33		6.60		4.65		6.82		—		—		4.74	
ADRs by PT	Incidence of ADRs by PT, events (%)													
Dysuria	3	(0.87)	9	(1.91)	0	—	9	(2.12)	0	—	0	—	12	(1.46)
Urinary retention	4	(1.17)	13	(2.77)	2	(4.65)	11	(2.59)	0	—	0	—	17	(2.07)
Residual urine volume increased	2	(0.58)	12	(2.55)	0	—	12	(2.82)	0	—	0	—	14	(1.70)

*Note:* MedDRA/J version 26.0.

Abbreviations: ADRs, adverse drug reactions; BPH, benign prostatic hyperplasia; PT, preferred term.

^a^ Only cases of male were counted.

Residual urine volume was measured in the 1031 patients in the SAS. Mean (± standard deviation) residual urine volumes at baseline and final evaluation were 20.5 ± 24.74 mL and 25.3 ± 43.80 mL, respectively, showing no significant increase (*p* = 0.143). Even in the seven patients with baseline residual urine volume ≥ 100 mL, mean residual urine volumes at baseline and final evaluation were 153.3 ± 59.76 mL and 121.1 ± 58.58 mL, respectively, showing no significant change (*p* = 0.218).

This survey also focused on cardiovascular AEs because of the potential risk of off‐target effects for β_1_ and β_2_ receptors. Cardiovascular AEs, which fell into PTs under the SOCs “cardiac disorders” or “vascular disorders,” or PTs narrowly defined under the standardized MedDRA queries (SMQs) “myocardial infarction,” “other ischemic heart disease,” or “cardiac failure,” was observed in nine patients (0.49%) in the SAS (Table 5). Of the 154 ADRs in 141 patients, four patients (0.16%) experienced cardiovascular ADRs including three patients with palpitations and one patient with congestive heart failure, which were all nonserious. Three of the four patients who developed cardiovascular ADRs had cardiovascular diseases as comorbidities. Two patients who developed palpitations had BPH and were treated with α_1_ blockers.

**TABLE 5:** Incidence of cardiovascular ADRs in patients with cardiovascular disease.

Cardiovascular disease status	No cardiovascular disease		With cardiovascular disease		Cardiovascular disease unknown		Total	
Patients, *n*	1288		516		44		1848	
Patients with ADRs, *n*	1		3		0		4	
ADRs, *n*	1		3		0		4	
Incidence of ADRs, %	0.08		0.58		—		0.22	
ADRs by PT	Incidence of ADRs by PT, events (%)							
Palpitations	1	(0.08)	2	(0.39)	0	—	3	(0.16)
Congestive heart failure	0	—	1	(0.19)	0	—	1	(0.05)

*Note:* MedDRA/J version 26.0.

Abbreviations: ADRs, adverse drug reactions; PT, preferred term.

Of 516 patients with cardiovascular disease as a comorbidity, three patients experienced cardiovascular ADRs (0.58%), while one patient in 1288 patients without cardiovascular disease reported cardiovascular ADR (0.08%).

### 3.3 Effectiveness

The effectiveness rate, or the ratio of cases where the overall improvement level was evaluated as “effective,” was 88.8% (1386 patients) in 1561 patients of the EAS.

As a result of statistical analysis, sex, age, presence or absence of previous OAB medications, and presence or absence of lower urinary tract obstructive disease had a significant effect on the effectiveness rate (*p* < 0.05). Even among patients with low effectiveness rates such as males, 85 years and older, with previous OAB medications, or with lower urinary tract obstructive disease, the effectiveness rates were still 83.8%–86.5%. The longer the administration period, the higher the effectiveness rate. High effectiveness was observed with long‐term use for 52 weeks or more (Table 6).

**TABLE 6:** Effectiveness rate by background factors.

			*n* (%)	Patients judged “effective” (%)	*p*
Patients in the effectiveness analysis set (EAS)			1561 (100.0)	1386 (88.8)	
Sex	Male		677 (43.4)	574 (84.8)	Fisher's exact test *p* < 0.001
	Female		884 (56.6)	812 (91.9)	
Age category 1	< 54 years		173 (11.1)	159 (91.9)	Cochran–Armitage test *p* = 0.010
	≥ 55, < 65 years		178 (11.4)	160 (89.9)	
	≥ 65, < 75 years		446 (28.6)	406 (91.0)	
	≥ 75, < 85 years		614 (39.3)	534 (87.0)	
	≥ 85 years		150 (9.6)	127 (84.7)	
Age category 2	< 65 years		351 (22.5)	319 (90.9)	Fisher's exact test *p* = 0.178
	≥ 65 years		1210 (77.5)	1067 (88.2)	
Age category 3	< 75 years		797 (51.1)	725 (91.0)	Fisher's exact test *p* = 0.006
	≥ 75 years		764 (48.9)	661 (86.5)	
Prostate volume (males only)	< 20 mL		18 (2.7)	14 (77.8)	Cochran–Armitage test *p* = 0.746
	≥ 20, < 30 mL		142 (21.0)	122 (85.9)	
	≥ 30, < 40 mL		102 (15.1)	88 (86.3)	
	≥ 40, < 50 mL		45 (6.6)	39 (86.7)	
	≥ 50 mL		56 (8.3)	48 (85.7)	
	Unknown		314 (46.4)	263 (83.8)	
Previous OAB medication	No		1191 (76.3)	1074 (90.2)	Fisher's exact test *p* = 0.001
	Yes		351 (22.5)	294 (83.8)	
	Unknown		19 (1.2)	18 (94.7)	
	β3‐adrenoceptor agonist	No	1292 (83.8)	1166 (90.2)	Fisher's exact test *p* < 0.001
		Yes	250 (16.2)	202 (80.8)	
	Anticholinergic agents	No	1413 (91.6)	1254 (88.7)	Fisher's exact test *p* = 0.884
		Yes	129 (8.4)	114 (88.4)	
Comorbidities	No		539 (34.5)	484 (89.8)	Fisher's exact test *p* = 0.443
	Yes		986 (63.2)	871 (88.3)	
	Unknown		36 (2.3)	31 (86.1)	
Comorbidities category	Lower urinary tract obstruction disease	No	1129 (74.0)	1016 (90.0)	Fisher's exact test *p* = 0.020
		Yes	396 (26.0)	339 (85.6)	
	Cardiovascular disease	No	1086 (71.2)	958 (88.2)	Fisher's exact test *p* = 0.242
		Yes	439 (28.8)	397 (90.4)	
Comorbidities details	Benign prostatic hyperplasia (males only)	No	272 (40.7)	229 (84.2)	Fisher's exact test *p* = 0.659
		Yes	396 (59.3)	339 (85.6)	
Concomitant medications	No		591 (37.9)	529 (89.5)	Fisher's exact test *p* = 0.675
	Yes		954 (61.1)	846 (88.7)	
	Unknown		16 (1.0)	11 (68.8)	
Concomitant medications category	α1‐antagonists	No	1218 (78.8)	1088 (89.3)	Fisher's exact test *p* = 0.426
		Yes	327 (21.2)	287 (87.8)	
	Phosphodiesterase (PDE) 5 inhibitors	No	1489 (96.4)	1333 (89.5)	Fisher's exact test *p* = 0.003
		Yes	56 (3.6)	42 (75.0)	
	Anticholinergic agents	No	1450 (93.9)	1296 (89.4)	Fisher's exact test *p* = 0.087
		Yes	95 (6.1)	79 (83.2)	
Concomitant medications details	Tamsulosin hydrochloride	No	1418 (91.8)	1270 (89.6)	Fisher's exact test *p* = 0.025
		Yes	127 (8.2)	105 (82.7)	
	Silodosin	No	1426 (92.3)	1269 (89.0)	Fisher's exact test *p* = 1.000
		Yes	119 (7.7)	106 (89.1)	
	Naftopidil	No	1472 (95.3)	1308 (88.9)	Fisher's exact test *p* = 0.565
		Yes	73 (4.7)	67 (91.8)	
	Tadalafil	No	1489 (96.4)	1333 (89.5)	Fisher's exact test *p* = 0.003
		Yes	56 (3.6)	42 (75.0)	
	Dutasteride	No	1491 (96.5)	1330 (89.2)	Fisher's exact test *p* = 0.182
		Yes	54 (3.5)	45 (83.3)	
	Fesoterodine fumarate	No	1513 (97.9)	1351 (89.3)	Fisher's exact test *p* = 0.018
		Yes	32 (2.1)	24 (75.0)	
Combination treatment	No		1371 (87.8)	1228 (89.6)	Fisher's exact test *p* = 0.005
	Yes		182 (11.7)	150 (82.4)	
	Unknown		8 (0.5)	8 (100.0)	
Combination treatment details	Pelvic floor exercises	No	1455 (93.7)	1293 (88.9)	Fisher's exact test *p* = 0.509
		Yes	98 (6.3)	85 (86.7)	
	Bladder training	No	1464 (94.3)	1310 (89.5)	Fisher's exact test *p* < 0.001
		Yes	89 (5.7)	68 (76.4)	
Administration period of vibegron	< 2 weeks		20 (1.3)	12 (60.0)	Cochran–Armitage test *p* < 0.001
	≥ 2, < 4 weeks		81 (5.2)	54 (66.7)	
	≥ 4, < 12 weeks		225 (14.4)	174 (77.3)	
	≥ 12, < 26 weeks		290 (18.6)	245 (84.5)	
	≥ 26, < 52 weeks		203 (13.0)	185 (91.1)	
	≥ 52 weeks		742 (47.5)	716 (96.5)	

Abbreviation: OAB, overactive bladder.

**FIGURE 2:**
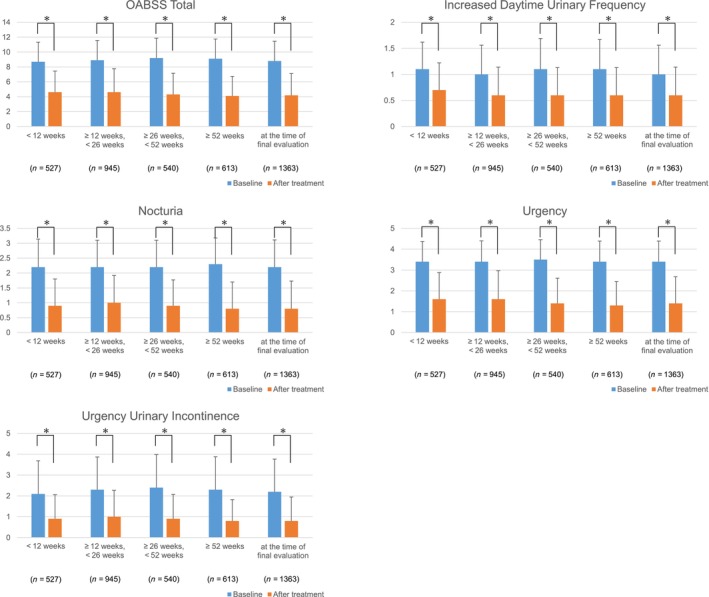
Change from baseline in OABSS total and subscore. *p < 0.001 (Wilcoxon signed‐rank test), Compared with baseline in each observation period. OABSS, overactive bladder symptom score.

Of the EAS, increased daytime urinary frequency, nocturia, urgency, and urgency urinary incontinence were evaluated in 1363 patients using OABSS at both baseline and each observation period. A significant decrease in OABSS total and subscore was observed at all the observation periods compared to the baseline (Figure 2). The amount of change from baseline in the OABSS total score was −4.1 to −5.1, indicating a decrease in score that far exceeded the MCIC.

**FIGURE 3:**
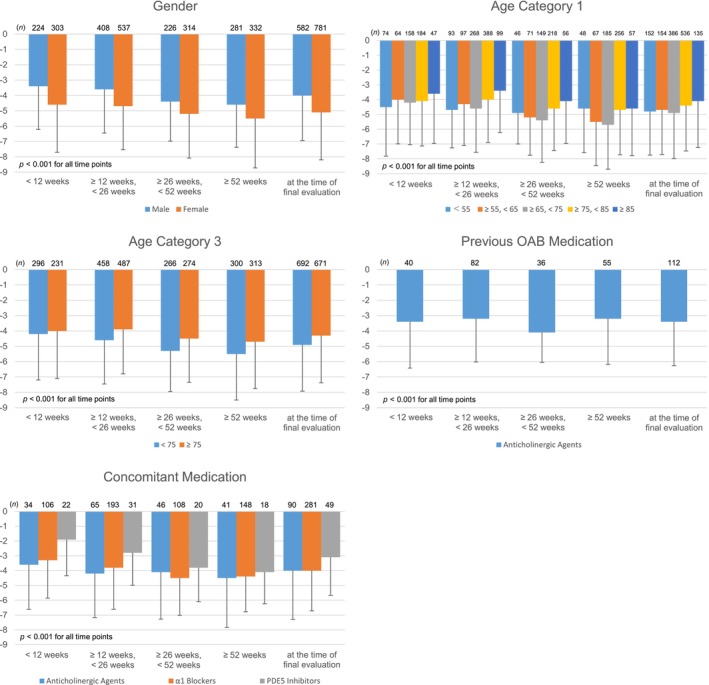
Change from baseline in OABSS total score in subgroups. *p* < 0.001 (Wilcoxon signed‐rank test), Compared with baseline in. each observation period. OABSS, overactive bladder symptom score.

Subgroup analysis was carried out by sex, age category 1, age category 3, use of anticholinergic agents as previous OAB medications, and use of anticholinergic agents, α_1_ blockers, or PDE5 inhibitors as concomitant medications, which significantly influenced the effectiveness rate. Significant score reductions were observed at all observation periods compared to baseline in all subgroups (Figure 3).

The MCIC achievement rate of the 1363 patients who performed OABSS evaluation was 70.21% (370 patients) at less than 12 weeks, 71.01% (671 patients) at 12 weeks or more but less than 26 weeks, 79.81% (431 patients) at 26 weeks or more but less than 52 weeks, the rate was 80.26% (492 patients) at 52 weeks or more, and 75.35% (1027 patients) at the final evaluation.

**FIGURE 4:**
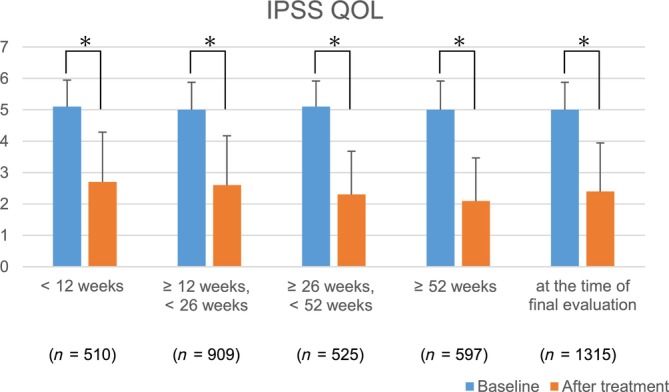
Change from baseline in IPSS QOL score in subgroups. *p < 0.001 (Wilcoxon signed‐rank test), Compared with baseline in. each observation period. IPSS QOL, International Prostate Symptom Score‐Quality of Life.

In the 1315 patients of the EAS whose IPSS QOL scores were evaluated at the same time points as the OABSS assessments, a significant decrease in IPSS QOL scores was observed at all the observation periods compared to the baseline (Figure 4).

## 4 Discussion

Vibegron was launched in Japan in November 2018. This survey was conducted to evaluate the safety and effectiveness of vibegron in Japanese patients with OAB in a real‐world clinical setting. In particular, we focused on the safety and effectiveness of vibegron in patients with BPH, about which very few studies have reported.

Baseline characteristics of the SAS (*n* = 1848) show that 822 patients (44.5%) were male, and 1423 patients (77.0%) were 65 years old or over. β_3_‐Adrenoceptor agonists (16.7%, 304 patients) and anticholinergic agents (9.2%, 168 patients) were used as previous OAB medications. The persistence rate at 52 weeks was relatively high at 42.5% (785 patients), which was common to a report that the persistence rate of mirabegron, another β_3_‐adrenoceptor agonist, was higher than anticholinergic agents [26]. The good persistence rate in the survey is thought to reflect the favorable benefit–risk balance of vibegron. In addition, the survey included 470 patients (57.2% of male patients) with BPH, 516 patients (28.6%) with cardiovascular disease, 390 patients (21.3%) taking α_1_ blockers, 112 patients (6.1%) taking anticholinergic agents, and 67 patients (3.7%) taking PDE5 inhibitors, for whom information was limited in clinical trials [16].

Of the 1848 patients in the SAS, 154 ADRs were reported in 141 patients (7.63%), which was almost the same incidence of 7.6% with vibegron 50 mg in the Phase 3 study in Japan [16]. The common ADRs were residual urine volume increase (1.30%), constipation (1.14%), dysuria and urinary retention (0.97%, each), cystitis (0.65%), and dry mouth (0.32%), which were all listed in the label when vibegron was released. No new events or signals affecting the safety of vibegron, even in long‐term use for more than 52 weeks, were observed.

AEs related to urinary retention, which is an important identified risk in the Japanese risk management plan of vibegron, occurred in 57 patients (3.08%), although urinary retention‐related AEs were not common in the Phase 3 study in Japan [16]. The risk of urinary retention‐related AEs was higher in males, patients ≥ 65 years old, patients with prostate volume ≥ 50 mL, patients with BPH, and patients using α_1_ blockers or PDE5 inhibitors. Those findings suggested that the larger proportion of male patients, elderly patients, and patients with BPH in this survey compared to Phase 3 study trials would have contributed to the higher incidence of urinary retention‐related AEs. On the other hand, no significant increase from baseline was observed in the mean residual urine volume of all patients for whom residual urine volume was measured or for patients with baseline residual urine volume ≥ 100 mL. Although there is no change in residual urine volume on average, it should be noted that residual urine volume would be increased in a certain number of patients and some of them would even develop urinary retention.

The main reason why the incidence of urinary retention‐related AEs is higher in men is that the presence of BPH is considered to be a major factor in developing them. This could explain the gender difference in the incidence of urinary retention‐related AEs.

In general practice, treatment for lower urinary tract obstructive disease is given priority over the treatment for OAB. In this survey, 43 of 470 BPH patients were not being treated for BPH, but the incidence of urinary retention‐related AEs was 4.65% (2 patients), lower than the 6.82% (29 patients) in the 425 patients being treated for BPH. BPH itself carries a risk of urinary retention as a complication [27]. Because the risk of urinary retention among BPH patients may be high regardless of treatment for BPH, it is important for healthcare professionals to closely monitor the voiding conditions or the residual urine volume during follow‐up visits and instruct patients to seek immediate medical attention if they notice any sign of difficulty urinating, particularly for patients with obstructive diseases of the lower urinary tract such as BPH.

β_1_ and β_2_ receptors are known to be expressed in cardiovascular tissue, raising concerns about potential off‐target effects of β_3_‐adrenoceptor agonists on cardiovascular tissue. In this survey, four of the 154 ADRs in the SAS were cardiovascular events (0.16%) and three of the 516 patients with concomitant cardiovascular disease experienced cardiovascular ADRs. In the Japanese postmarketing survey of mirabegron, the incidence of cardiovascular ADRs was reported as 0.48%, and a network meta‐analysis comparing the safety of mirabegron and vibegron found that only mirabegron showed a higher risk of cardiovascular events [28, 29]. Vibegron is reportedly more selective for β_3_ receptor and less specific for β_1_ and β_2_ receptors than mirabegron [30, 31]. Previous studies, including this survey, have not shown any major concerns regarding off‐target effects of vibegron related to β_1_ and β_2_ receptors.

The effectiveness rate in the EAS was 88.8% (1386/1561 patients). A high effectiveness rate was also seen in patients with BPH or on concomitant α_1_ blockers or anticholinergic agents. An effectiveness rate > 80% was demonstrated in patients switching from β_3_‐adrenoceptor agonists or anticholinergic agents, just as some studies have reported that vibegron is effective after switching from those medications [32, 33].

A significant decrease in OABSS scores was observed in all observation periods compared to baseline. The amount of change in OABSS total score from baseline was −4.1 to −5.1, indicating a decrease in score far in excess of the MCIC. In subgroup analyses by sex, age, pretreatment use of anticholinergic agents, and concomitant use of anticholinergic agents, α_1_ blockers, or PDE5 inhibitors, significant reductions in score were observed in all observation periods compared to baseline in all subgroups. Vibegron is considered highly useful even in patients ≥ 85 years old, with BPH, or receiving treatment in combination with anticholinergic agents, α_1_ blockers, or PDE5 inhibitors.

A potential limitation of the survey was that it was based on a survey of real‐world patients and was therefore neither placebo‐controlled nor blinded.

## 5 Conclusions

This survey identified no major concerns regarding the safety and effectiveness of vibegron in Japanese OAB patients, except for urinary retention‐related events in male patients with BPH. Vibegron is considered to be a useful drug for treating OAB symptoms in a real‐world clinical setting.

## Conflicts of Interest

The authors declare no conflicts of interest.

## Data Availability

Due to the nature of this research, supporting data are not available.
